# CellMigrationGym: a data-driven framework utilizing deep reinforcement learning to unravel mechanisms of cell migration

**DOI:** 10.1186/s12919-026-00365-5

**Published:** 2026-02-20

**Authors:** Dali Wang, Jiawei Yang, Zi Wang, Yichi Xu, Zhirong Bao

**Affiliations:** 1 Department of Electric Engineering and Computer Science, University of Tennessee, University of Tennessee, Knoxville, 37996 TN USA; 2 Developmental Biology Program, Sloan Kettering Cancer Center, Sloan Kettering Cancer Center, New York, 20001 NY USA; 3 Biological and Environmental Sciences, Oak Ridge National Laboratory, Oak Ridge National Laboratory, Oak Ridge, 37831 TN USA

**Keywords:** Cell migration, Cell structure identification, 3-D live image, Deep reinforcement learning, OpenAI gym, PyBullet

## Abstract

Cell migration is a fundamental phenomenon in biology that underlies normal development as well as cancer. Recently, a data-driven approach was introduced that uses deep reinforcement learning(DRL) and 3-D live images to study cell migration. This approach formulates the cell migration process as a sequential Markov decision process (MDP), so that hypotheses of the underlying mechanism of the observed migration can easily be incorporated as high-level regulatory rules and constraints for DRL. The application of the approach successfully uncovered a novel mechanism of cell migration in C. elegans embryogenesis that involves a modular organization of cells by using ubiquitous labels of cell nuclei and simple rules based on empirical statistics of the images. This success demonstrates new opportunities to use DRL to infer the biology of cell migration without prior knowledge. This paper presents an open framework, CellMigrationGym, to standardize the DRL approach to study cell migration. Built upon common packages (OpenAI Gym, PyBullet, and DRL libraries), CellMigrationGym provides powerful and flexible functions to investigate cell migration behavior. Through a case study, we demonstrate the critical functions of CellMigrationGym with technical details, such as 1) preparation and standardization of multiple observational data, 2) reward formulation and DRL model configuration appertaining to the hypotheses of migration mechanism (such as gradient-driven and collective cell behavior-driven mechanisms), 3) exploration of migration scenarios under hypothesized mechanisms, and 4) evaluation of neighboring cell’s influence on the cell migration.

## Introduction

Cell migration, the directed movement of a single cell or a group of cells in response to chemical and/or mechanical signals, is a fundamental cellular process that occurs throughout whole organism life, from embryonic development until death. For example, in a developing embryo, cell migration is the driving factor for various morphogenetic events. While in adult organisms, cell migration occurs during vital cellular processes such as tissue renewal and repair [1, 2].

Understanding the regulatory mechanisms behind cell migration is of essential significance since it contributes to multiple substantial pathological processes in embryos or organisms. Computer tools have been developed to explore the coordination, regulation, and interaction between the migrating cell and its neighboring cells, as long as their microenvironment.

**Fig. 1:**
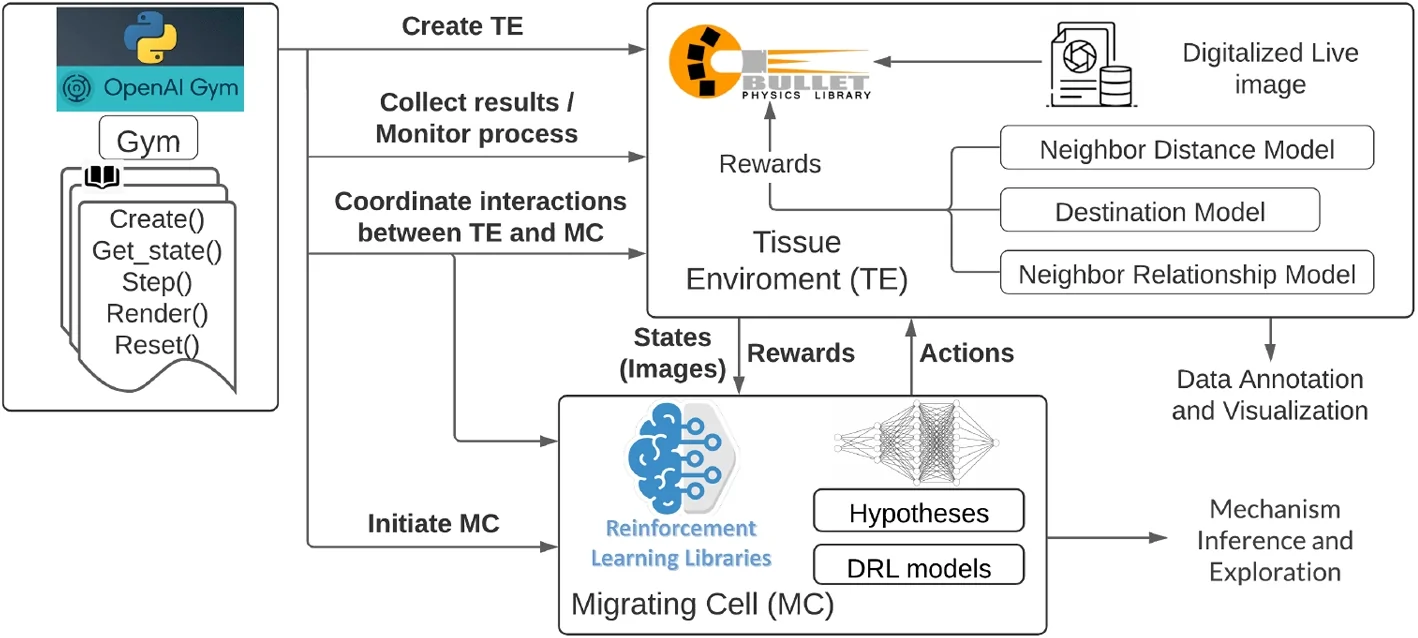
Architecture of CellMigrationGym with three key components: tissue environment, migrating cell, and OpenAI Gym

### Methods for cell migration modeling

Physical models have been developed to understand cell migration and its biological significance [3, 4]. Early models adopted differential equations to describe the physical principles in cell protrusion, cell adhesion, and retraction [5]. These models successfully explained several typical cell migration in 2-D scenarios, but generally failed to capture the complexities of cell migration in 3-D environments [6].

Agent-based modeling (ABM) [7, 8] became a suitable simulation tool for cell migration study in complicated environments. In an ABM system, each cell is represented as an agent, interacting with each other as well as the environment. Predefined rules encoded with inherited biological information are used for controlling the behaviors of the cells. The rules can either be known biological regulatory mechanisms, or be statistical observations extracted from experimental measurements when specific mechanisms are missing. Among the earlier ABM-related approaches, Cellular Potts Model (CPM) [9, 10] was a good example that was adopted to effectively simulate certain kinds of cell behavior. Setty et al. presented models on *C. elegans* germ line [11] and mouse pancreas [12] with ABM and statechart [13]. The fact that their simulation of ligand interaction distance, developmental regulation of cell cycle, and pancreatic organogenesis show highly consistent with the biological experimental result, proves the validity of this approach.

These models made positive progress and created meaningful results. However, they were limited by the understanding of the regulatory mechanisms during the development process. Nowadays, with the abundance of experimental and observational datasets, we need innovative, data-driven tools to explore novel regulatory mechanisms of cell migration.

### DRL for cell migration study

Recently, a new approach was developed to use DRL within an ABM for cell migration modeling using 3-D live images [14]. It modeled the migrating cell as a DRL agent interacting with an embryo that was modeled as the environment. It was the first time that the cell migration process was reformulated as a sequential Markov decision process (MDP). Hypotheses of the underlying mechanism of the observed migration were incorporated as high-level regulatory rules and constraints for DRL.

Furthermore, the comparison between the observational data and the DRL results was used to infer the validity of the proposed high-level regulatory rules on cell migration. To further infer cell-cell interactions and collective cell behaviors as potential underlying migration mechanism, Hierarchical DRL (HDRL), known for multiscale learning and data efficiency, was adopted to examine cell migrations based on images with ubiquitous nuclear labels and simple rules formulated from empirical statistics of the images [15]. When applied to *C. elegans* embryogenesis, HDRL revealed a multi-phase, modular organization of cell movement that was confirmed by imaging experiments as an underlying migration mechanism. These works proved the DRL as a powerful, data-driven approach to inferring biology without prior knowledge.

This study presents an open framework, CellMigrationGym, to standardize and expedite the DRL approach to study cell migration and infer the underlying biology. The innovations of CellMigrationGym are manifested in several ways: 1) adopts an open framework design that supports live-cell imaging; 2) provides essential functions for cell migration investigation; 3) enables easy hypothesis formulation of migration mechanism and rapid DRL model configuration; and finally 4) exhibits the novel use of DRL to study dynamic biological processes.

## Method

### Microscopic datasets

Live-cell imaging is the study of living cells using time-lapse microscopy. After the pioneer study in the beginning of the 21st-century [16], several microscopy methods have been developed to obtain a better understanding of cellular dynamics, such as cell migration, cell development, and intracellular trafficking [17].

Our study focuses on datasets from fluorescent live imaging where synthetic and organic fluorescent stains are applied to label specific proteins or organic chemical compounds [18]. These datasets have been used to understand the complex machinery of a cell or entire organisms. Some examples are the applications of live-cell images to study the development of *C. elegans*, fruit fly, zebrafish, and mouse [19–22].

### Architecture of CellMigrationGym

CellMigrationGym contains several key components, a Tissue environment (with reward formulation), Migrating cells (with mechanism hypothesis and DRL model), and OpenAI Gym [23] (Fig. 1).

***Tissue Environment: ***The tissue environment is essentially an agent-based model that is built based on PyBullet [24]. Digitized live images are required as input data to form a 3-D tissue environment, where the positions of cells are identified and cells are traced between frames. The name, location, and size of each cell are collected in digitized image files. The tissue environment extracts and collects information about each cell’s name, location, size, and its children’s names. The environment also calculates the rewards for a specific action of the migrating cell.

***Reward Formulation: ***Reward is formulated to reflex three fundamental relationships of the migrating cell: 1) neighbor cells, 2) the spatial distribution of the migrating cell and its neighbors, and 3) the distance to the destination. Specifically, the reward in the Tissue Environment is a sum of feedback from three Models: Neighbor Relation Model, Neighbor Distance Model, and Destination Model. The Neighbor Relationship Model determines the neighbor relationship between any cell pairs. The Neighbor Distance Model evaluates the spatial distribution between the migrating cell and its neighbor cells. The Destination Model assesses the distance relationship between the migrating cell and the target cell.

***Migrating Cell:*** Each migrating cell is treated as an RL agent in CellMigrationGym. It uses DRL models to determine its action based on the feedback from the tissue environment. The action usually is a single movement (speed and direction) that the migrating cell can take at each timestep.

***Mechanism Hypotheses and DRL Model Configuration:*** Inside the migration cell, users propose migration hypotheses and then configure DRL models to incorporate the hypothesis into CellMigrationGym.

***OpenAI Gym:*** OpenAI Gym toolkit is used to coordinate and monitor the interactions between the Tissue environment and Migration cells. Specifically, CellMigrationGym uses OpenAI Gym to create a connection with the Tissue environment *Create()*, initial the migration cells as RL agents, monitor the Tissue environment *Get_state()*, coordinate the stepwise interactions between the Tissue environment and Migrating cell *step()*, visualize the simulation results *render()*, and reset the whole environmental parameters *reset()*.

### Data visualization, annotation, and manipulation

**Fig. 2:**
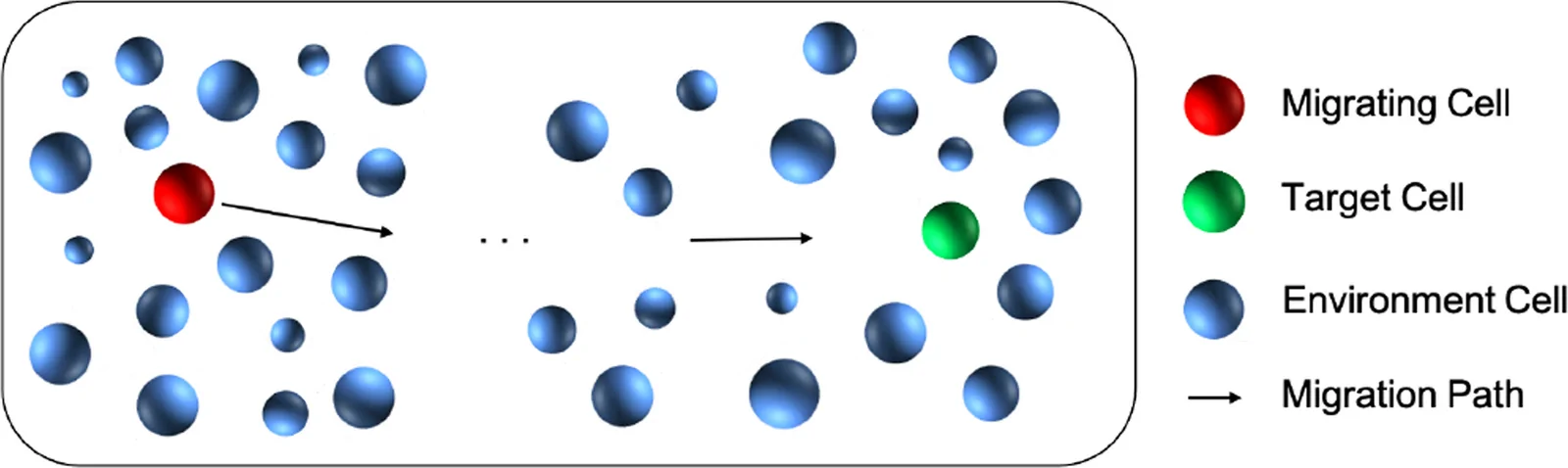
Example input image for CellMigrationGym. The migration path is shown for better illustration

CellMigrationGym provides rich functions for data annotation, manipulation, and visualization. It uses live images to setup its 3-D environment, in which each cell is represented as a sphere with its own position, size, and color. Figure 2 illustrate an example of the 3-D environment, in which the size and position of cells are configured with the microscopic data. Three types of cells (a migrating cell, environment cells, and a target cell) are annotated with different colors.

**Fig. 3:**
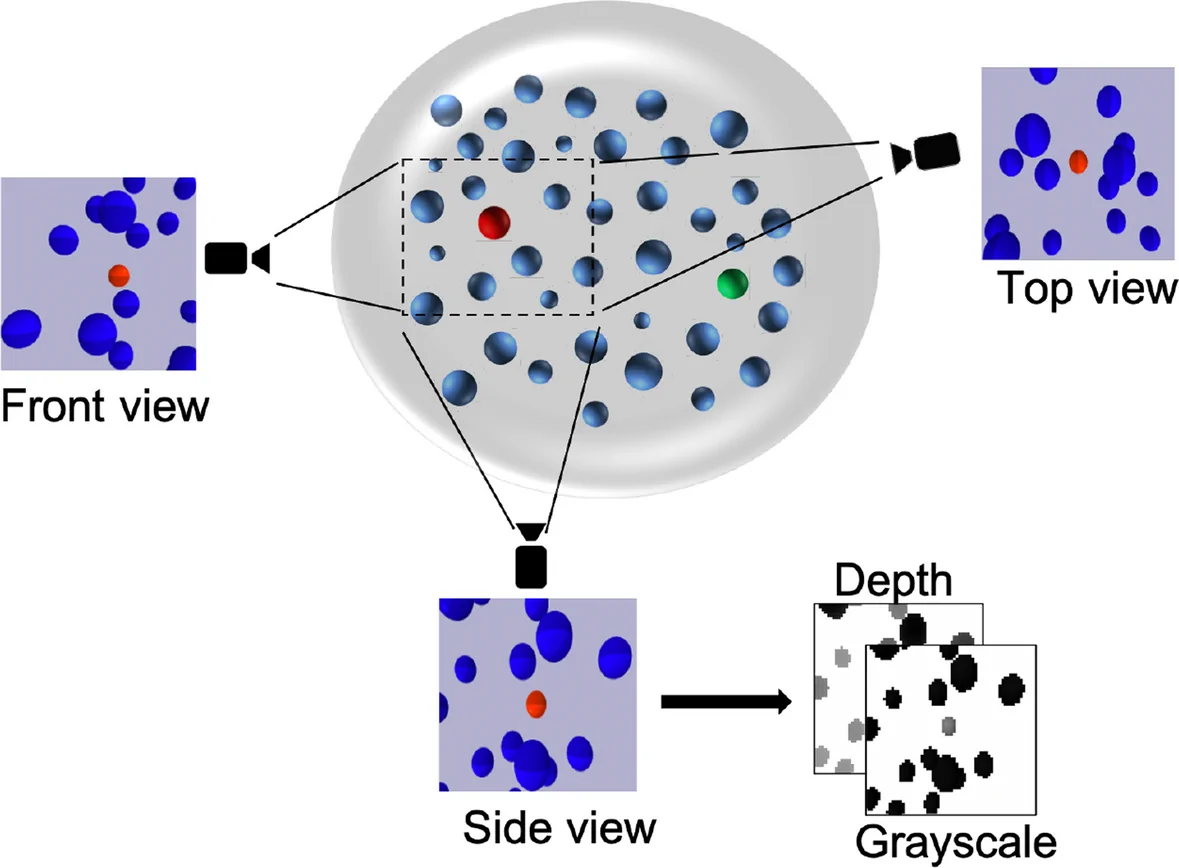
Example perspective views during the migrating process. Red sphere represents the migrating cell, blue sphere represents environment cell

CellMigrationGym allows users to change the perspective view of the migrating process in different places by setting the camera target position, angle, and distance to the target position. The camera perspective view can also be changed interactively with the mouse and keyboard during the simulation (Fig. 3). Moreover, within CellMigrationGym, cells can be selected with their names, colored in different colors, and tracked their movement during the migrating process. Each cell’s position and rotation can be tracked or changed at different timestep. CellMigrationGym can generate various kinds of images, such as 3-D views, 2-D views, and depth images, to meet the needs of DRL experiments.

## Result and discussion

We use a case study to demonstrate the critical functions of CellMigrationGym with technical details. The migration process we examined is known as “Cpaaa intercalation” in the early stage of *C. elegans*’ development. Cpaaa (migrating cell) is a cell born at the dorsal posterior side of the embryo and the intercalation happens when the Cpaaa moves between two rows of ABarp cells. The Cpaaa intercalation starts around 15 minutes after the birth of Cpaaa and ends when Cpaaa becomes the neighbor of the ’ABarpaapp’ cell (target cell).

We provide technical details of CellMigrationCell setup using digitized live image data, input image generation, and observation data standardization. Most importantly, we present the workflow of migration mechanism investigation under two migration hypotheses. The first mechanism is called “influence by gradient”, where we assume that Cpaaa migration is driven by a strong chemical gradient between Cpaaa and the target cell. While under the second mechanism, Cpaaa is assumed to be driven by collective cell behavior during migration. We focus on how to formulate the rewards using the three basic models within the Tissue Environment, and how to configure the DRL models to test these hypotheses.

### 3-D time-lapse live imaging data

In a total of fifty wild-type embryos, three embryos are used for computational experiments (RL training), and six are used for validation. These live images are obtained and processed with a Zeiss AxioObserver Z1 inverted microscope frame with Zeiss 40x objective and the MetaMorph software (Molecular Devices). The resolution of the images on the X, Y, Z, axes are 0.254 µm, 0.254 µm, and 0.1 µm, respectively. There are 30 planes used in the Z direction. Starrynite II [25] and AceTree [26, 27] are used for cell lineage tracing and manual correction of the traced lineage. All the data are stored in AceTree data format [26].

### CellMigration setup

#### Tissue environment

We read the digitized live image data (in AceTree data format) and convert them into NumPy [28] array format in python. The location and nucleus size data of each cell are used to form the 3-D Tissue environment. Cells other than the Cpaaa (migrating cell) in the embryo are environment cells, among which the ABarpaapp is marked as the target cell (migration destination). For each embryo at each timestep, we extract and stored each cell’s location (x,y,z), nucleus size, and cell name into a NumPy array. Since the original nucleus data has a large timestep of 60 seconds, a 10 fold upsampling interpolation is linearly performed to shrink each timestep into 6 seconds in order to train the migrating cell (RL agent) more accurately. The reward is a sum of feedback from the three following models.

***Neighbor Relationship Model: *** A Neighbor Relationship Model for *C. elegans* embryos was developed [29] that uses a set of handcrafted features (including cells’ locations, sizes, and the total number of cells in the embryo) to determine whether a neighbor relationship exists between a cell pair. The neighbor relationship model is implemented with a random forest classifier and trained with the data of *C. elegans* wild-type embryos and the ground truth is obtained with the Voronoi algorithm. During each timestep of the training process, the model finds all direct neighbors of the migrating cell as a neighbor group.

***Neighbor Distance Model: *** The model evaluates the distance relationship between the migrating cell and its neighbors found by the Neighbor Relationship Model. The model obtains a single distance-based reward $R_{Ni}$ by calculating the ratio between the distance of the two cells and the sum of their radius. The radius of each cell is estimated based on the cell lineage identity, embryo volume, and the total number of cells in the embryo. More details are presented in the previous work [14, 15, 30]. In this study, a single distance-based negative reward $R_{Ni}$ ranges from (−1,0) is given linearly when the ratio increases within an acceptable range. Based on the statistics (mean, deviation, distance to the egg shell, and total number of cells) calculated with the cells’ distances from 50 wild-type embryos [8, 29], the acceptable ratio ranges from (0.3,0.8). The statistical measurements we used include mean distance, deviation, distance between each cell to the eggshell, and total number of cells in the embryo, as well as the distribution of the generations of the cells in the embryo. If the ratio is too small (less than 0.3 in this case), a negative reward of −1000 is given. If the ratio is larger than 0.8, no negative reward is given. The distance reward $R_N$ is the sum of all single distance reward $R_{Ni}$.

***Destination Model: *** The model provides feedback (rewards) when the migrating cell becomes a stable neighbor of the target for 5 timesteps. A reward of 100 is given when the migrating cell reaches the final destination within a given timestep (90% of the observational data migrating time). This model is flexible and can be modified and reconfigured to represent specific migration regulatory rules. More information in Migration mechanism exploration section.

#### Migrating cell

Cpaaa is modeled as an RL agent, which uses its DRL model to determine its action based on the feedback from the tissue environment. The direction of the action is one of the eight directions that Cpaaa can move at a single timestep. The step size of the action is calculated by using the average speed of the migrating cell plus or minus 20% randomness with a normal distribution. The average speed is derived from the observation data by dividing the total distance (between the migrating cell and its target at the beginning of the migration) with the total migration time.

**Fig. 4:**
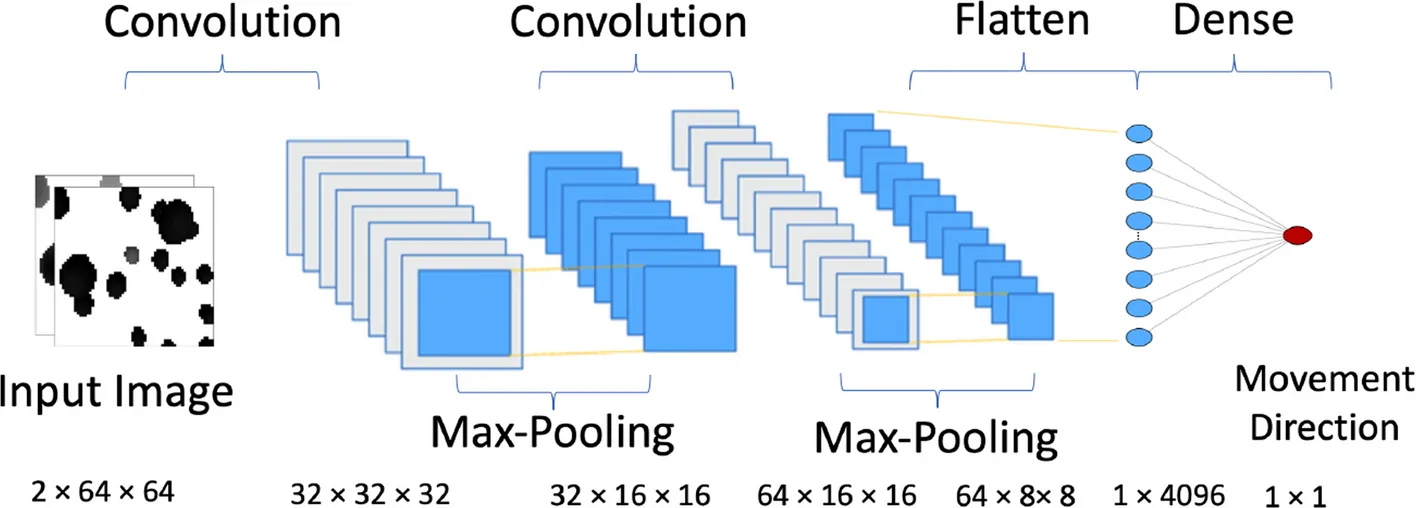
Policy network of the Cpaaa cell

***DRL Model Configuration under Two Migration Hypotheses: *** The workflow of Cpaaa migration mechanism investigation using CellMigrationGym is quite flexible. To better explore the first proposed mechanism (influence by gradients), a single-level DRL model (i.e., deep Q-learning (DQN) [31, 32]) is adopted in Cpaaa to learn its movements. Technically, the policy network of Cpaaa contains a CNN that has two convolutional layers (which extracts feature vectors from the input at each timestep) and a fully connected layer (Fig. 4).

To better represent the second proposed mechanism that Cpaaa is driven by collective cell behavior during migration, we introduce the subgoals that are environment cells among a possible Cpaaa migration path. We use a two-level Hierarchical DRL model (i.e., hierarchical deep Q-learning (H-DQN) [33]. The higher-level module learns and picks appropriate subgoals to guide the migrating cell to the final destination. It uses a fully-connected network (with 3 hidden layers of 512, 1024, and 1024 nodes) to select a potential subgoal out of the secondary neighbors based on the current position of Cpaaa. The policy network of the lower-level module is the same as Fig. 4, which learns proper actions for the migrating cell to reach the subgoal/final destination.

#### Input images for DRL models

**Fig. 5:**
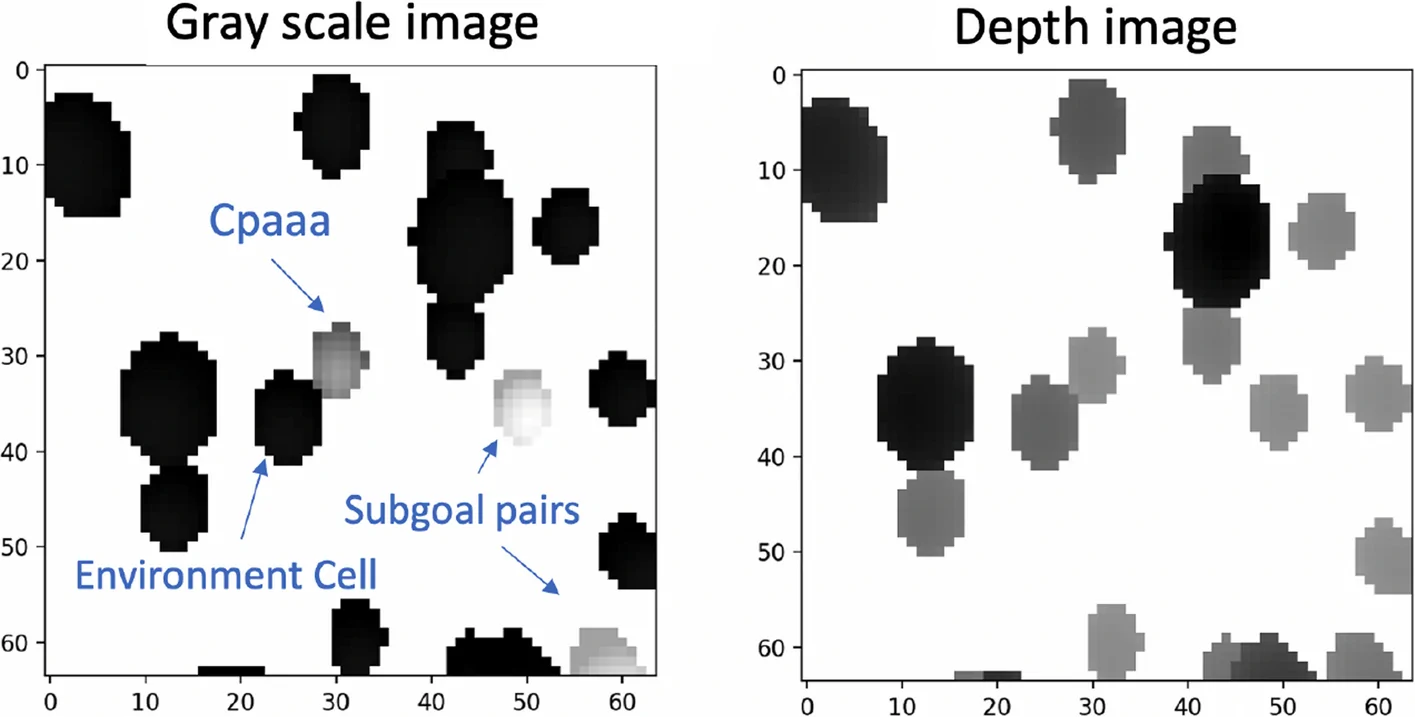
Example input images for the RL training of the migration cell. The subgoal is only used for collective cell behavior scenarios

CellMigrationCell is versatile of creating perspective views, in our study, we use a 2-D top-view image and a depth image to represent the 3-D environment for fast RL training of the migrating cell. Specifically, at each timestep, we first capture and extract an RGB image $(3 \times 64 \times 64)$ and a depth image (64×64). Then we convert the RGB image into a grayscale image (64×64) to reduce the complexity of the input. The grayscale image contains the (*x*, *y*) location and size information of each cell, and the depth image provides the z-axis information of each cell. Finally, we concatenate the grayscale image and the depth image as the input image $(2 \times 64 \times 64)$ for RL training of the migrating cell. Example input images are shown in Fig. 5.

**Fig. 6:**
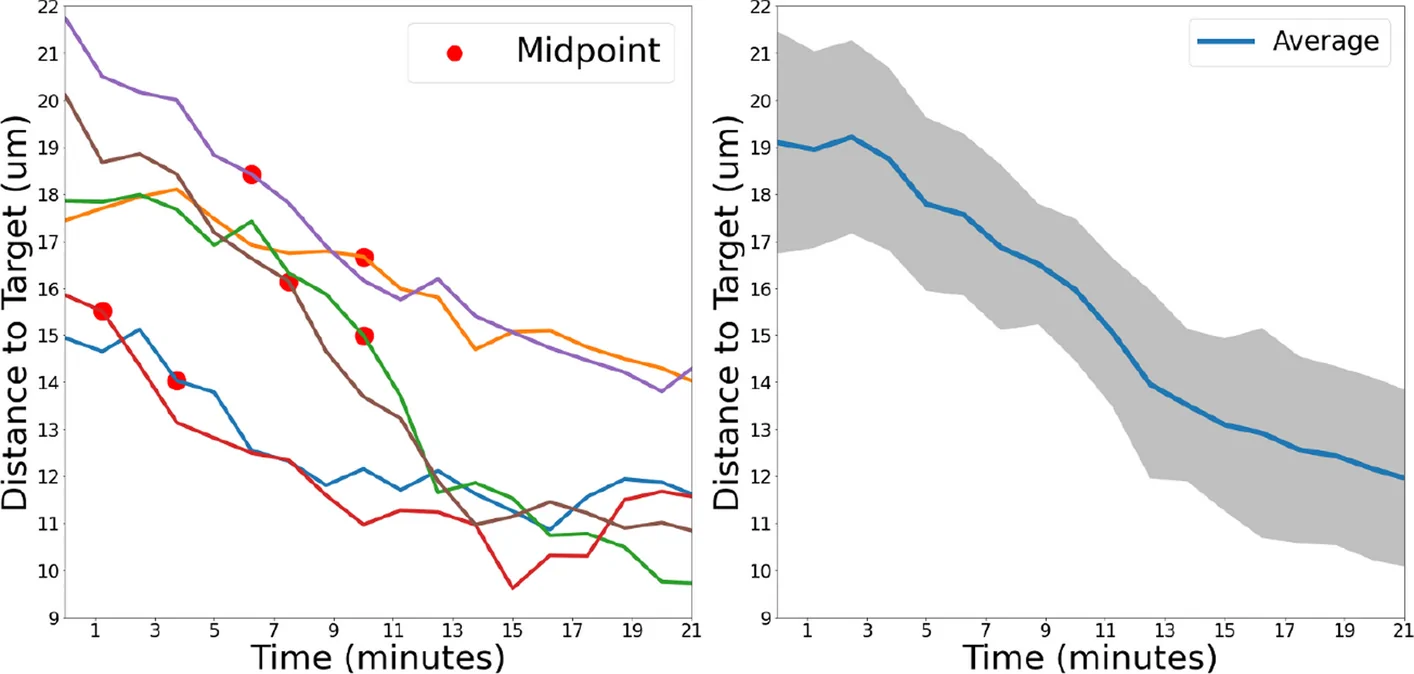
Position of the Cpaaa (distance to the target cell) during the migration, obtained from the observational data of six embryos

**Fig. 7:**
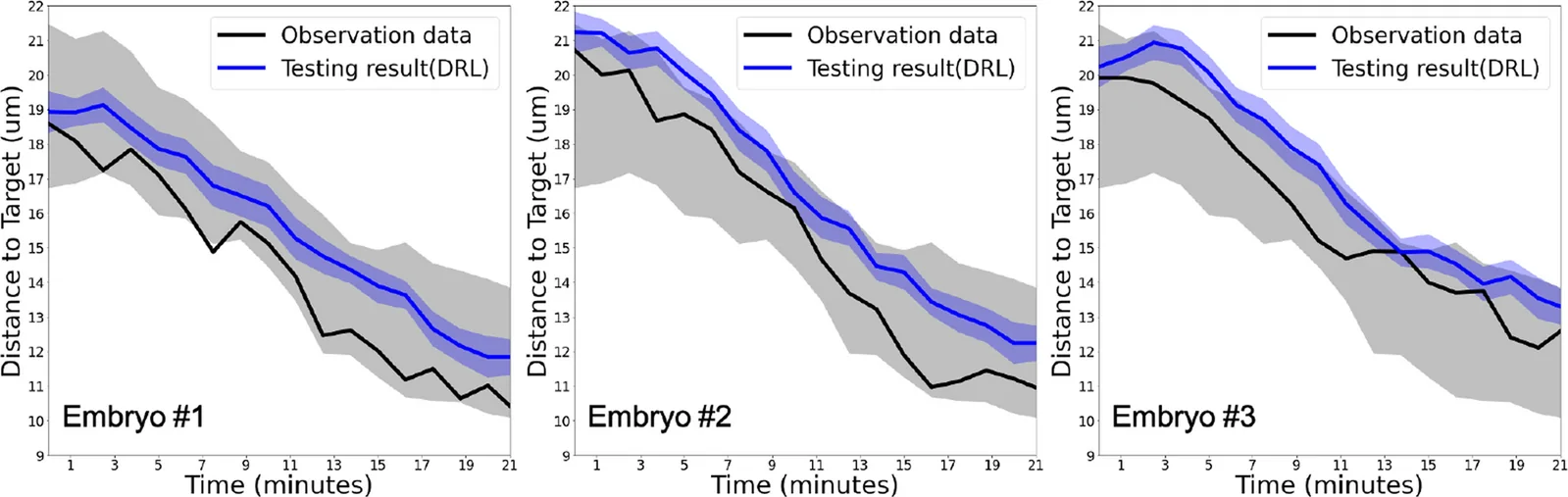
Position of the migrating cell (distance to the target cell) during Cpaaa intercalation influenced by gradient in three embryos

**Fig. 8:**
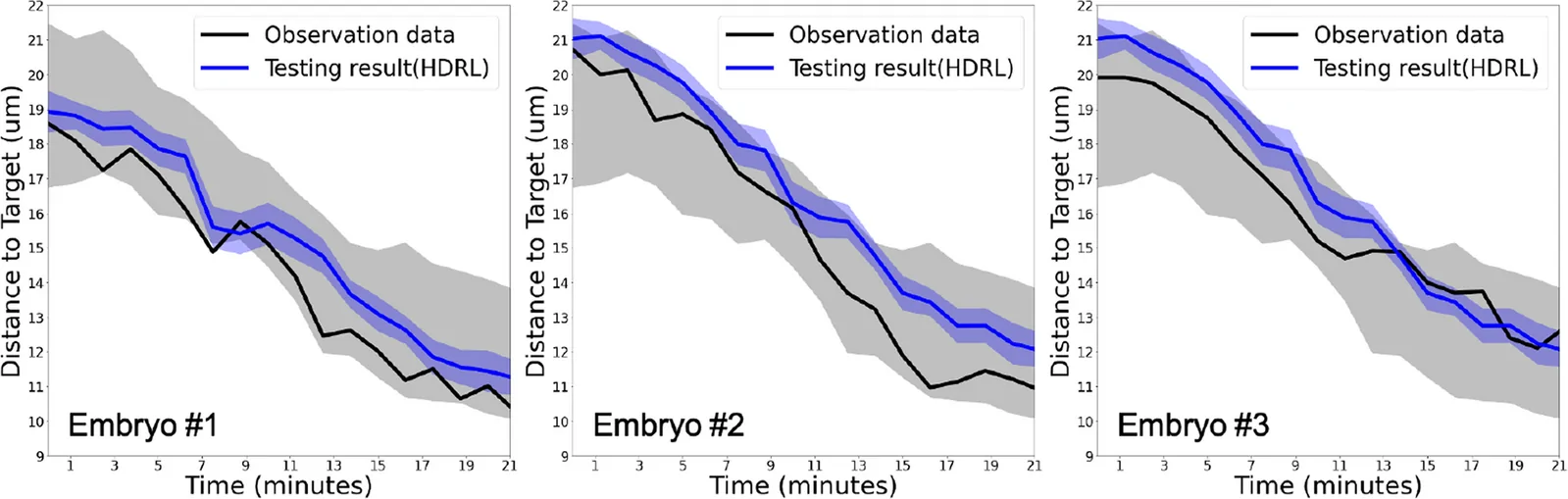
Position of the migrating cell (distance to the target cell) during the Cpaaa intercalation driven by collective cell behavior in three embryos

### Multiple observational data preparation

The live images of *C. elegans* embryos contain a large data disparage associated with individual embryo growth. For example, Cpaaa in different embryos has different migrating and dividing times. In order to better handle the data variations among different observational datasets, we develop a new approach to standardize the data.

We first extract all the necessary data including each cell’s location (*x*, *y*, *z*), nucleus size, and cell name for each embryo. Then we record the migrating process time obtained from the intersection of the starting and dividing time of the migrating cell and the target cell. Next, we calculate the distance array that represents the distance between the migrating cell and the target cell at each timestep during the migrating process. We align the distance array of different embryos using a Midpoint to calculate the average distance array and its standard deviation. The Midpoint is calculated by using the closest point to the average of the maximum and minimum in the distance array. Finally, we plot the average distance array with a shaded region where the upper and lower bound of the region are obtained by one standard deviation of the average distance array using observational data from six embryos. The resulting plot of the distance between Cpaaa and its target cell during the migration is shown in Fig. 6. The left panel shows the distance change during migration using the original observational data. The right panel shows the distance change during migration using the standardized data. The blue line represents the average distance array, while the gray shaded region indicates one standard deviation using the data of six embryos.

### Migration mechanism exploration

#### Migration under the influence of gradients

In this case, we investigate whether the migrating mechanism of Cpaaa is caused by the influence of the gradient between the Cpaaa and its target cell. We assign the destination model with an additional gradient-based reward function that is proportional to the distance between the Cpaaa and the target cell. Therefore, in this case, the destination model provides not only a sparse and delayed reward at the end of the migration (when Cpaaa reaches the target cell), it also continuously provides additional dense feedback (rewards) during the Cpaaa intercalation. Other information on the CellMigrationCell setup, input image generation, and the configuration of the tissue environment, and the policy network of the migrating cell are the same as detailed before. A total of three embryos are used during the DRL training (via cross-training). It is also worth noting that CellMigrationGym provides a more flexible and unified way for DRL experiments than the previous framework [14].

We adopt an DQN model along with an Adam optimizer (with $\beta _1$ = 0.9, $\beta _2$ = 0.999), a batch size of 64, and a learning rate of 0.0001. Moreover, a target network is included to stabilize the training process and it is updated every 1000 iterations. The network is trained for 900 epochs with an ϵ-greedy factor initialized to 0.3 and gradually increases to 0.95. The total training process using a Nivida DGX station takes around 5 hours.

Figure 7 shows the distance between Cpaaa and the target cell during the Cpaaa intercalation in three wide-type embryos. The blue line represents the averaged distance change collected from five simulations. The black line is the distance calculated from the observational data. Blue shaded regions indicate one standard deviation of the five simulation results. For better illustration, the grey region from Fig. 6 is also plotted to show the variance of the observational data.

#### Migration driven by collective cell behavior

We setup an H-DQN model to investigate whether Cpaaa intercalation is driven by the collective cell behavior. We introduce the subgoals that are environment cells among a possible Cpaaa migration path. Whenever a subgoal is achieved, that is when Cpaaa forms a stable neighbor relationship with the subgoal cells, the Destination model provides an intermediate reward. In this case, the Destination model only provides delayed and sparse rewards when the subgoals or final destination are achieved. The use of subgoals reduces the search space and the sample size [34] for Cpaaa training. Technically, for fast and stable learning, each subgoal has a pair of cells that are chosen from secondary neighbors (neighbor of a neighbor) of the migrating cell using the neighbor relationship model.

The higher-level module uses a fully-connected network to select a potential subgoal out of the secondary neighbors based on the current position of Cpaaa. When a subgoal is selected by the higher-level module, we use the annotation function of CellMigrationGym to color the subgoal cells in white and generate input image pairs (2-D grayscale image plus depth image) for the lower-level module. The lower-level module learns proper actions for the migrating cell to reach the subgoal/final destination. The batch size is 32 and the ϵ-greedy factor is set to 0.8 initially, and gradually grows to 0.95. The rewards for achieving a subgoal and the final destination are set as 10 and 100 respectively.

The migrating cell is successfully trained (with three embryos) to reach the destination using CellMigrationGym. The total training time of 900 epochs using the DGX is around 7 hours. Again, this application proves that CellMigrationGym is a powerful framework for DRL experiments, superior to the previous framework [15].

Figure 8 shows the distance between Cpaaa and the target cell during the Cpaaa intercalation in the wide-type embryos. The blue line represents the averaged distance change collected from five simulations. The blue line is the distance calculated from the observational data. Blue shaded regions indicate one standard deviation of the five simulations.

### Mechanism verification with live imaging

Results in Fig. 7 indicate that the influence of gradient can be a possible underlying migrating mechanism for Cpaaa intercalation. However, no explicit physical and chemical evidence was found in biology experiments to approve the existence of gradient-based elements that drive Cpaaa towards the target.

**Fig. 9:**
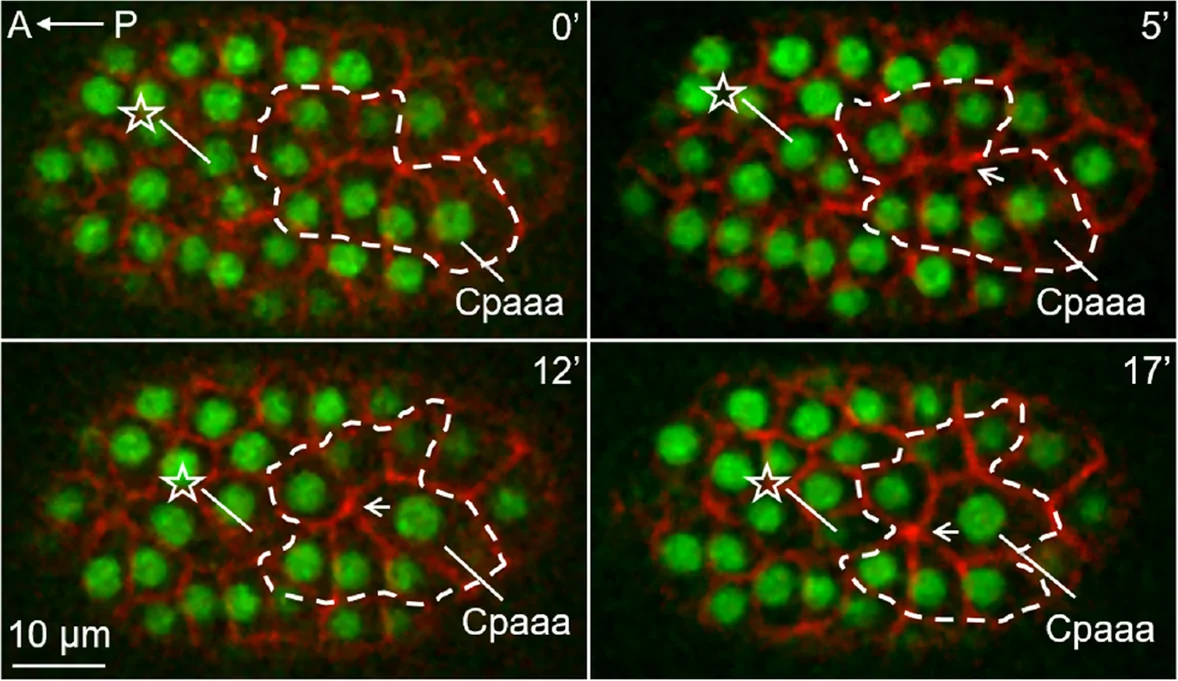
Cpaaa intercalation with sequential rosettes. Star: ABarpaapp (target cell); Arrow: rosette center; Dashed line: Cpaaa and six neighbor ABarp cells; Three rosettes form at approximately minute 5, 12 and 17 minutes after Cpaaa starts to migrate (minute 0: 18 mins after the birth of Cpaaa)

Figure 8 also indicates that the collective cell behavior could be the underlying migration mechanism. Different from the previous DRL experiment that lacks supporting biology observation, we find supportive evidence for cell-cell interactions in this case. Figure 9 reveals a collective behavior of Cpaaa and its neighboring cells to mediate its migrations. Specifically, Cpaaa forms a sequence of multicellular rosettes with the neighbor ABarp cells during its migration (the dashed region in Fig. 9). Rosettes are defined as five or more cells joined together at a common point of contact. Three rosettes form over time with sequential edge contraction and resolution events (arrowheads in Fig. 9). The formation and resolution of each rosette are correlated with Cpaaa movement anteriorly towards the ABarpaapp cell (star in Fig. 9). In this study, a series of multicellular rosettes including Cpaaa and its neighbors are formed to guide Cpaaa towards the target, which is a novel cell movement mechanism and we name it as sequential rosettes [15].

### Evaluation of neighbor cell’s impact on migration

**Fig. 10:**
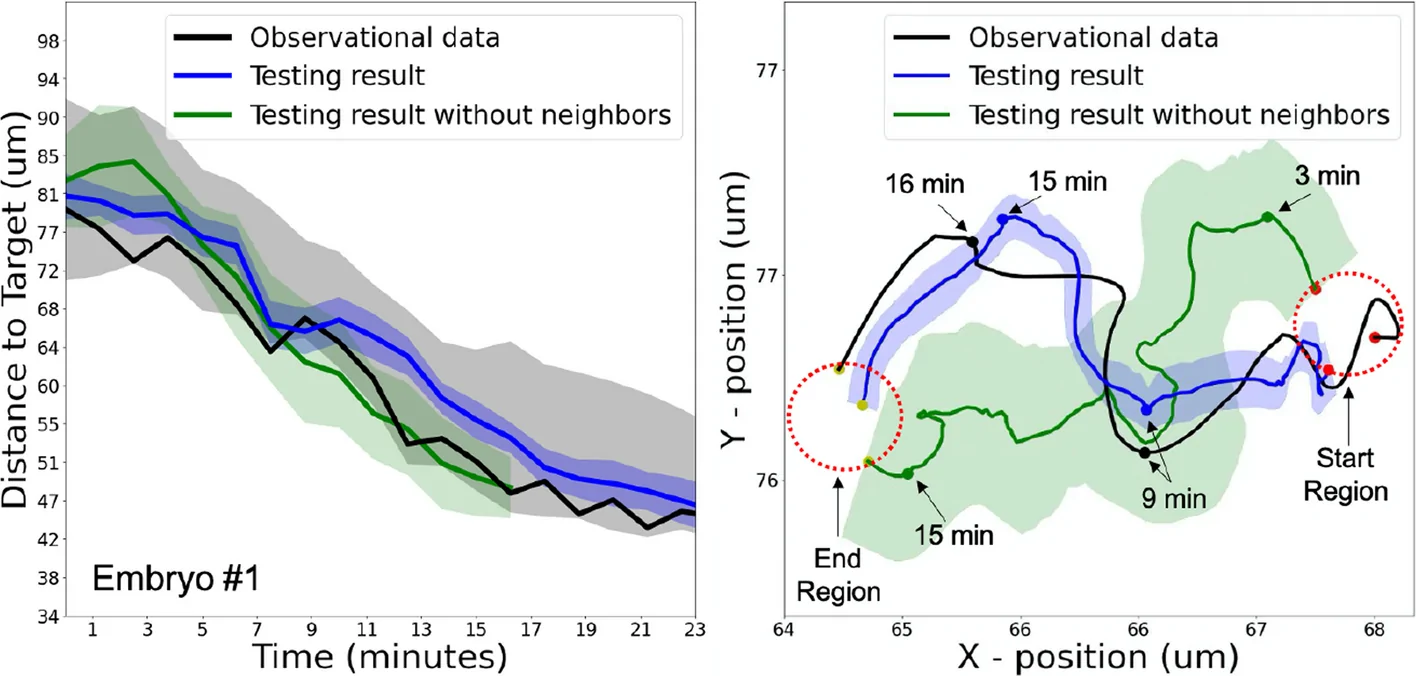
Position of the migrating cell (distance to the target cell) (left) and migration paths from a 2-D top-view (right) in the embryo #1. Shaded regions indicate one standard deviation/spread of five ensemble runs

We use CellMigratinGym to further evaluate the impact of neighbor cells on the Cpaaa migration. We use the Neighbor Relationship Model to determine the neighbor cell during Cpaaa migration and then remove these neighbor cells to generate the input images for Cpaaa training. Then we compare the distance to the target cell collected from the DRL experiments (with or without neighbor cells). Figure 10 shows the distance between Cpaaa and the target cell) (left) and the top-view of the migration path (right) in the embryo #1. As shown in the left panel, with the neighbor cells, Cpaaa’s path (blue) matches well with the observational data (black). When there are no neighbor cells (green), Cpaaa migrates much faster and reaches the destination 5 minutes earlier than the observed arriving time. The right panel further reveals the differences. With the presence of neighbor cells, the Cpaaa migration path matches with the observational data in terms of start/end region, general shape, and key turning points. On the other hand, after the removal of neighbor cells, the migration path (green) is very different from the observed path and comes with a much larger variance. We conclude that the neighbor plays an important role during the migration process. Similarly, We can use CellMigrationGym to explore the influence of individual cell(s) on the migration path of the migrating cell.

## Conclusion

We presented a powerful framework, CellMigrationGym, to standardize and expedite the application of DRL to investigate cell migration behavior. Compared with these previous models, such as the Cellular Potts Model and traditional Agent-based models, our approach provides new ways to understand and explore the regulatory mechanisms for cell migration during the development process. CellMigrationGym provides essential functions to allow users not only to explore migration mechanisms with DRL but also to evaluate individual cells’ influence on cell migration. We also used a case study to demonstrate the flexible use of CellMigrationGym to investigate cell migration under different mechanism hypotheses. We hope the open framework CellMigrationGym can inspire advanced applications of DRL to study other dynamic biological processes, such as proliferation patterns, gene expression dynamics, or neuronal activities.

## Data Availability

Data and the source code is available in Github.
